# Regulation of assimilate import into sink organs: update on molecular drivers of sink strength

**DOI:** 10.3389/fpls.2013.00177

**Published:** 2013-06-04

**Authors:** Saadia Bihmidine, Charles T. Hunter, Christine E. Johns, Karen E. Koch, David M. Braun

**Affiliations:** ^1^Division of Biological Sciences, University of MissouriColumbia, MO, USA; ^2^Interdisciplinary Plant Group, University of MissouriColumbia, MO, USA; ^3^Missouri Maize Center, University of MissouriColumbia, MO, USA; ^4^Horticultural Sciences Department, University of FloridaGainesville, FL, USA; ^5^Plant Molecular and Cellular Biology Program, University of FloridaGainesville, FL, USA

**Keywords:** carbohydrate partitioning, kernel, maize, sink strength, sorghum, stem, sucrose, sugarcane

## Abstract

Recent developments have altered our view of molecular mechanisms that determine sink strength, defined here as the capacity of non-photosynthetic structures to compete for import of photoassimilates. We review new findings from diverse systems, including stems, seeds, flowers, and fruits. An important advance has been the identification of new transporters and facilitators with major roles in the accumulation and equilibration of sugars at a cellular level. Exactly where each exerts its effect varies among systems. Sugarcane and sweet sorghum stems, for example, both accumulate high levels of sucrose, but may do so via different paths. The distinction is central to strategies for targeted manipulation of sink strength using transporter genes, and shows the importance of system-specific analyses. Another major advance has been the identification of deep hypoxia as a feature of normal grain development. This means that molecular drivers of sink strength in endosperm operate in very low oxygen levels, and under metabolic conditions quite different than previously assumed. Successful enhancement of sink strength has nonetheless been achieved in grains by up-regulating genes for starch biosynthesis. Additionally, our understanding of sink strength is enhanced by awareness of the dual roles played by invertases (INVs), not only in sucrose metabolism, but also in production of the hexose sugar signals that regulate cell cycle and cell division programs. These contributions of INV to cell expansion and division prove to be vital for establishment of young sinks ranging from flowers to fruit. Since INV genes are themselves sugar-responsive “feast genes,” they can mediate a feed-forward enhancement of sink strength when assimilates are abundant. Greater overall productivity and yield have thus been attained in key instances, indicating that even broader enhancements may be achievable as we discover the detailed molecular mechanisms that drive sink strength in diverse systems.

## INTRODUCTION

Whole-plant carbohydrate partitioning is the process by which the products of atmospheric carbon dioxide assimilation are exported from leaves through the veins to distant non-photosynthetic tissues. Researchers aiming to improve crop yield have focused on two key aspects of whole-plant carbohydrate partitioning: enhancing carbohydrate production in leaves (i.e., increasing source capacity) and/or improving the utilization of photoassimilates by sink organs (i.e., enhancing sink strength). Examples of strategies to enhance carbohydrate production include attempts to increase light interception either by increasing the number of leaves or total leaf area, breeding for stay-green traits, and enhancing photosynthesis by improving the capacity of the plant to fix carbon (Sakamoto et al., 2006; Hammer et al., 2009; Zheng et al., 2009; Zhu et al., 2010; Raines, 2011; Ruan et al., 2012a). In regards to the strategy of enhancing sink strength, researchers are attempting to increase the number, size, and carbohydrate-storing activity of sink organs (Ho, 1988; Marcelis, 1996; Herbers and Sonnewald, 1998; Smidansky et al., 2002). Sink strength has been defined as the competitive ability of a sink organ to import photoassimilates, and depends on both its physical (size) and physiological (activity) capabilities (Ho, 1988; Marcelis, 1996; Herbers and Sonnewald, 1998).

Carbon fixation occurs in source leaves and is biochemically driven by photosynthesis. The fixed carbon, primarily in the form of sucrose, moves from mesophyll cells into the phloem, where it is transported long-distance through veins into sink tissues. Carbohydrates assimilated during the day in excess of leaf export capacity are transiently stored as starch in chloroplasts and as sucrose in the vacuole, and then remobilized during the night to continuously supply sink tissues (Rhoades and Carvalho, 1944; Kaiser and Heber, 1984; Smith and Stitt, 2007; Slewinski et al., 2008; Eom et al., 2011). The imported carbon is then either directed into immediate usage in metabolic processes important for growth and development or stored inside sink organs (Gifford and Evans, 1981). In source leaves, assimilated carbon can move into the phloem entirely through a symplastic (cytoplasmic) path, or it may first be exported to the apoplast (cell wall space) surrounding the phloem, prior to being imported into phloem companion cells and/or sieve elements (Lalonde et al., 2004; Braun and Slewinski, 2009). In the symplastic pathway, transport of sugar occurs cell-to-cell through plasmodesmatal connections, passively flowing down a concentration gradient. In contrast, in the apoplastic path, the distribution of fixed carbon can move against a concentration gradient in an energy-dependent process. In this case, sugar transporters import sugars from the phloem apoplast across the cell membrane, resulting in its accumulation to high levels in the cytoplasm (Turgeon, 2006; Ayre, 2011). Consumption and storage of sugar can be controlled at various points along the transport path. These regulatory points include phloem unloading from vascular tissues into storage parenchyma cells, compartmentation at the tonoplast of storage parenchyma cells, and metabolism and/or storage within sink tissues (Patrick, 1997). Additionally, diverse environmental factors can also affect sink strength (Marcelis, 1996; Andersen, 2003; Aranjuelo et al., 2011). In this review, we discuss recent findings related to the molecular drivers of sink strength in stems, seeds, flowers, and fruits. For recent progress discussing source strength and phloem loading in leaves, see Lalonde et al. (2004), Turgeon (2006), Sauer (2007), Ma et al. (2008), Braun and Slewinski (2009), Kühn and Grof (2010), Slewinski and Braun (2010), Ainsworth and Bush (2011), Ayre (2011), Baker et al. (2012), Braun (2012), and Chen et al. (2012).

## THE STEM AS A STORAGE ORGAN

In plants, storage organs are structures that accumulate carbohydrates derived from photosynthesis. They include fruits, seeds, roots, and/or stems. An example of stem acting as a storage organ is the potato (*Solanum tuberosum*) tuber, which is a modified stem capable of storing large amounts of starch (Wiltshire and Cobb, 1996). Other examples are found in grasses, such as sweet sorghum (*Sorghum bicolor*) and sugarcane (*Saccharum officinarum*; Slewinski, 2012). As members of the Panicoideae subfamily, both plants are C_4_ species that can accumulate high concentrations of fermentable sugars, mainly sucrose, in their stems. Because of the high stem sucrose contents, both plants are models for strong stem sinks, and are being used for the production of ethanol as a biofuel (Waclawovsky et al., 2010; Calviño and Messing, 2012). These plants will be the focus of the first part of the review.

In both sweet sorghum and sugarcane, photoassimilates are first used for plant growth and development during early vegetative stages. Afterward, when the internodes (the sections of the stem between two nodes) have elongated, stems transition to sugar storage organs, where most of the accumulated carbon is stored as sucrose (Hoffmann-Thoma et al., 1996; Lingle, 1999; Almodares et al., 2008; Almodares and Hadi, 2009; Calviño and Messing, 2012; Slewinski, 2012). It has been proposed that stored sugars in the stem are used to buffer photoassimilate supply to the grains during plant growth and development. Both sweet sorghum and sugarcane stems exhibit a developmentally progressive increase in sucrose content in which they accumulate higher concentrations of sucrose in more mature internodes (i.e., lower down the stem) compared to younger ones (i.e., upper; Batta and Singh, 1986; Hoffmann-Thoma et al., 1996; McCormick et al., 2006; Tarpley and Vietor, 2007; Slewinski, 2012).

Even though sweet sorghum and sugarcane are closely related and both accumulate large amounts of sucrose in their stems, there are similarities and differences in regards to their sucrose accumulation. In both cases, there is a developmental decrease in the activity of sucrose metabolizing enzymes, particularly acid invertases (INV) as stem elongation nears completion (Ruan et al., 2010). Later, however, the radial transfer of sucrose from the phloem into the storage cells of the mature internodes may follow a different path in these two grasses (see below). Evidence indicates the presence of distinctive mechanisms and the possible involvement of different molecular and physiological drivers underpinning the accumulation of high levels of sucrose in sweet sorghum and sugarcane stems.

Sweet sorghum cultivars brought to the United States, mainly from China and Africa, were subject to selection and improvement for disease resistance, high stem sugar content, stalk erectness, and biomass accumulation (Reddy et al., 2005; Rooney et al., 2007). Because of this selection-based bottleneck effect, little variation is found within cultivated sweet sorghum varieties, especially in terms of stem sugar composition (Ali et al., 2008; Murray et al., 2009). Likewise, since the major focus in sugarcane improvement programs has been to increase stem sugar content, the genetic variation related to this trait in germplasm collections used in breeding programs is also limited (Aitken et al., 2008; Creste et al., 2010; Mancini et al., 2012). However, despite this narrow genetic variation in the sweet sorghum and sugarcane germplasms, the presence of subgroups with different sugar contents has been reported in each species. Two groups of sweet sorghum have been identified using simple sequence repeats (SSRs) markers: group IX with high sugar content and group VII with low sugar yield (Ali et al., 2008). Additionally, Murray et al. (2009) used both SSR and single nucleotide polymorphism (SNP) markers to genotype 125 cultivars of sorghum, and classified the sweet sorghum varieties into three major groups based on differing stem sugar contents. Similarly, using amplified fragment length polymorphism (AFLP) and SSR markers, two types of sweet sorghum with different brix content (a measure of the level of solutes in stem juice) were detected in a germplasm collection in Mexico (Pecina-Quintero et al., 2012). Likewise, differences were observed in sugar composition and content from eight sugarcane varieties, including four commercial cultivars (Tai and Miller, 2002). These findings suggest that genetic differences exist in the control of sink strength within each species, which should be considered when investigating the molecular drivers of sugar accumulation.

### SUGAR TRANSPORT AND UNLOADING INTO STEM PARENCHYMA CELLS

#### Transport path

**FIGURE 1:**
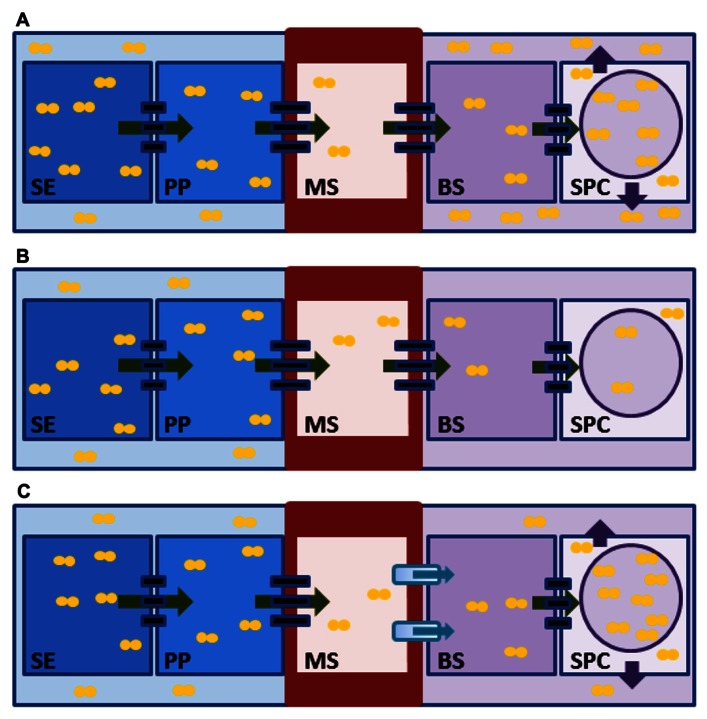
**Models for sucrose movement in sugarcane and sweet sorghum stems**. **(A)** In mature sugarcane stems, sucrose (orange circles) moves symplastically cell-to-cell (green arrows) through plasmodesmata (black rectangles) from the phloem sieve element (SE) to the stem parenchyma cell (SPC). In the SPC, some of the sucrose is stored in the vacuole (purple circle), and some is released to the apoplast (purple arrows) to increase the sink strength and sucrose storage capacity of the tissue. Sucrose in the SPC apoplast is prevented from diffusing back into the phloem apoplast by the suberized and lignified cell wall (thick red outline) of the mestome sheath cells (MS). **(B)** In immature sorghum stems, sucrose follows a symplastic path from the SE to the SPC cells. This tissue is actively growing and not storing much sucrose. **(C)** In ripening sorghum stems, sucrose movement from the SE to the SPC includes an apoplastic step. The suberized and lignified cell wall of the MS cell necessitates that the sucrose efflux must occur from either the MS or the bundle sheath cell (BS, not shown). Import could occur in either the BS or in the SPC (not shown). For simplicity, sucrose efflux and import are shown at the MS–BS interface by the shaded blue rectangle and blue arrow. The majority of the stored sucrose is intracellular. PP, phloem parenchyma cell.

Sugarcane and sweet sorghum may differ in their strategies for accumulating high levels of sucrose in their stems. In maturing sugarcane stems, sucrose transport follows a predominantly symplastic pathway when moving from the phloem into the storage parenchyma cells (**Figure 1A**; Lingle, 1989; Moore and Cosgrove, 1991; Rae et al., 2005; Patrick et al., 2013). This is supported by evidence that the sap collected from the xylem of sugarcane stems, which is contiguous with the phloem apoplast, contains virtually no sucrose (Welbaum et al., 1992), and by the observation that a fluorescent dye loaded into leaves is unloaded from the stem phloem and able to move symplastically through plasmodesmata to the stem parenchyma cells (Rae et al., 2005). In the stem, as sucrose begins to accumulate, some of it is effluxed to the apoplast of stem parenchyma cells by turgor-driven homeostatic leakage, and the apoplastic sucrose concentration can reach approximately 400–700 mM (Welbaum and Meinzer, 1990; Moore and Cosgrove, 1991; Welbaum et al., 1992; Patrick et al., 2013). Sucrose accumulation in the stem parenchyma cell apoplast serves as an additional storage compartment and increases sink strength. At maturity, when sucrose accumulates to very high levels in the stem apoplast, sucrose backflow into the phloem is prevented by the lignified and suberized cell walls of the inner, mestome sheath (a ring of thick-walled cells that surrounds the vein internally to the bundle sheath cells; Welbaum et al., 1992; Rae et al., 2005; Walsh et al., 2005; Patrick et al., 2013). This structural adaptation is also present in sorghum (Artschwager, 1948) and presumably functions to isolate the parenchyma cell apoplast from the phloem apoplast.

Sucrose movement in sorghum initially follows a symplastic path into the growing internode (**Figure 1B**), but is reported to later include an apoplastic path during internode ripening (**Figure 1C**; Tarpley et al., 1996; Tarpley and Vietor, 2007). This model is based upon the specific activity of stem-infused radiolabeled sucrose recovered from growing axillary branches vs. ripening internodes. Additional support for this scenario using alternative approaches, such as determining whether the sucrose transporters implicated in apoplastic transport (see below) are required to import sucrose across the plasma membrane of the stem parenchyma cells, is needed. Approximately 80–90% of the sugar in sorghum stem internodes accumulates intracellularly within the parenchyma cells, and only 10–20% of the sugar accumulates in the stem apoplast (Tarpley and Vietor, 2007). The five- to ten-fold difference in sucrose levels between the symplast and apoplast of stem parenchyma cells suggests that the mechanisms of sorghum and sugarcane sucrose storage may be distinct. However, two caveats to this model need to be considered. First, the differences reported in intracellular vs. apoplastic sucrose concentrations were measured using two grain sorghum lines rather than sweet sorghum materials, and therefore, genetic differences between these sorghum types could explain the observed differences from sugarcane. Second, it is possible that sorghum uses a similar mechanism of turgor-driven sucrose leakage to the apoplast in mature stem parenchyma cells. In their study, Tarpley and Vietor (2007) used ripening internodes for their measurements of sucrose compartmentation, which could reflect that the storage cells had not reached their maximal capacity and not yet effluxed sucrose to its final concentration in the apoplast. In support of this hypothesis, a previous analyses of sugar content using the same stem-infusion technique in two other sorghum varieties, comparing plants at the grain filling stage to those 2 weeks after grain maturity, showed that sucrose levels continued to increase in the apoplast free space compared to the intracellular compartment (Tarpley et al., 1996). Hence, the symplastic and apoplastic sucrose contents in sorghum stems at final maturity may not be as different from what was found for sugarcane. Further work is necessary to characterize the transport path and compartmentation of sucrose in sweet sorghum and to determine if it utilizes a different mechanism than sugarcane.

#### Sugar transporters

In sweet sorghum, biochemical studies have shown that sucrose must cross the plasma membrane and tonoplast for storage in the stem parenchyma cells, indicating the involvement of sugar transporters (Hoffmann-Thoma et al., 1996). Sucrose transporters (SUTs) are H^+^/sucrose symporters and are the most-studied class of transporters capable of translocating sucrose across a membrane (Lalonde et al., 2004; Sauer, 2007; Braun and Slewinski, 2009; Kühn and Grof, 2010; Ainsworth and Bush, 2011; Ayre, 2011). In the sorghum genome, there are six SUTs (SUT1–6) that have been proposed to encode proteins located in the plasma membrane and/or tonoplast (Braun and Slewinski, 2009).

The hypothesis that transporters are involved in high sucrose accumulation was recently examined in sweet sorghum. Transcriptional analyses revealed that SUT mRNAs, specifically SUT1 and SUT4, correlate with high sugar content in sweet sorghum stems when compared to grain sorghum stems (Qazi et al., 2012). Still, the exact roles of SUTs in sucrose accumulation in stem sink tissues remain undefined. In maize, SUT1 was found to be expressed in stems (Aoki et al., 1999); however, its function in stem tissues is unknown as it has only been shown to function in sucrose loading into the phloem in leaves (Slewinski et al., 2009, 2010). In sugarcane, using a peptide antibody raised against the orthologous SUT1 protein (ShSUT1), Rae et al. (2005) found that ShSUT1 was expressed in the mestome sheath and vascular parenchyma cells in stem and leaf tissues, suggesting SUT1 has non-phloem-loading functions in this species. The authors proposed a role for ShSUT1 in retrieving sucrose from the apoplast as a biochemical barrier to maintain the sucrose gradient between the phloem and stem storage parenchyma cells. Thus, it is possible that the SUT1 RNA expression detected in sweet sorghum stems (Qazi et al., 2012) reflects a similar role to the closely related ShSUT1. More work is needed to determine the function of SUT genes in stem sucrose partitioning.

Recently, other sugar transporter proteins, such as SWEETs and tonoplast monosaccharide transporters (TMTs), were shown to transport sucrose across membranes (Wingenter et al., 2010; Chen et al., 2012). However, the roles of these additional sucrose transporters in facilitating sucrose transfer into the stem have not been characterized (Ayre, 2011; Slewinski, 2011, 2012; Baker et al., 2012; Braun, 2012; Chen et al., 2012).

### SUGAR METABOLISM AND STORAGE IN THE STEM

Sucrose metabolism and storage processes in sink cells represent an important determinant of stem sink strength. During initial expansion of sweet sorghum and sugarcane stems, INVs contribute to sink strength and stem size. However, later, sucrose is stored in the large vacuoles of stem parenchyma cells. Very little of this stored sucrose is hydrolyzed during its transfer into the ripening stem (Lingle, 1989; Tarpley et al., 1996; Tarpley and Vietor, 2007). Further, after the stems have matured and elongation has ceased, these tissues show low metabolic activity (Tarpley et al., 1996), prompting the question as to whether sucrose metabolism remains an important driver of sink strength in maturing sweet sorghum and sugarcane stem tissues.

Several key enzymes involved in sucrose metabolism are INV, sucrose synthase (SUS), sucrose phosphate synthase (SPS), and sucrose phosphate phosphatase (SPP). INV enzymes catalyze the cleavage of sucrose into glucose and fructose, and various types of INV enzymes are found in plants and function in different locations, including the cell wall, vacuole, and the cytoplasm (Ruan et al., 2010; Vargas and Salerno, 2010; Patrick et al., 2013). SUS is another enzyme that cleaves sucrose into fructose and UDP-glucose in a reversible reaction (Winter and Huber, 2000; Patrick et al., 2013). Finally, SPS and SPP play an important role in sucrose synthesis in the cell. In the cytosol, SPS and SPP are jointly responsible for the irreversible synthesis of sucrose from UDP-glucose and fructose-6-phosphate (Lunn and MacRae, 2003).

Compared to sweet sorghum, grain sorghum accumulates little sucrose in the stem, and most of the carbohydrates produced are directed toward storage in seeds (Tarpley et al., 1994; Calviño and Messing, 2012; Qazi et al., 2012). This contrast suggests that sink strength differs in the seeds and stems between the two varieties. By examining sugar accumulation and storage in three cultivars of sweet sorghum, Hoffmann-Thoma et al. (1996) ascribed negligible roles for the three key sucrose metabolizing enzymes SUS, SPS, and INV. Similarly, Qazi et al. (2012) compared sugar accumulation between a sweet and a grain sorghum variety at three developmental stages in both the upper and lower internodes. They found that variety, stage, and internode position contributed to differences seen in sugar content. However, differences were not detected in the activities of SUS, SPS, and INV between the two varieties, indicating these enzymes are not likely to be primary contributors to the variation observed in stem sugar content.

In the sugarcane stem, sucrose accumulation has been linked to the differential RNA or protein expression or localization of enzymes involved in sucrose metabolism (Schafer et al., 2004; Grof et al., 2006). However, because of the complexities of stem tissues being composed of multiple cell types and the developmental progression in stem sucrose accumulation, correlations between expression and function may not always be evident. Nonetheless, in some cases, the activities of sucrose metabolizing enzymes are thought to regulate sucrose levels in sink tissues (Zhu et al., 1997; Botha and Black, 2000; Koch, 2004; Grof et al., 2007). For instance, soluble acid INV activity is low during maturation in varieties that store high levels of sugar (Zhu et al., 1997). Meanwhile, at least three SUS isoforms are present in sugarcane, and sucrose synthesis is correlated with SUS activity. In addition, the ratio of sucrose synthesis to breakdown is higher in mature internodes, and researchers have suggested that different isoforms of SUS are expressed in young internodes compared to mature ones (Schafer et al., 2004). A positive relationship between SPS activity and sucrose accumulation has also been demonstrated in sugarcane (Botha and Black, 2000; Grof et al., 2007). However, studies using enzyme activity and isotope tracers have demonstrated that sucrose can be transferred from phloem tissues into the vacuoles of storage parenchyma cells without being catabolized and then resynthesized in the stem (Lingle, 1989). Collectively, these data suggest that the enzymes involved in sucrose metabolism likely play a minor role in sucrose storage by the maturing stem and are unlikely to be major drivers of stem sink strength at later stages of development in sugarcane.

It thus appears that additional processes besides sucrose metabolizing enzymes may be important contributors to the high level of sucrose accumulation in sugarcane stems. In the case of sorghum, where quantitative trait loci (QTL) mapping is possible, researchers have identified genomic regions related to sucrose accumulation in the stem (Murray et al., 2008a,b; Ritter et al., 2008). However, the genes underlying these QTLs have yet to be identified. Transcriptional profiling experiments to characterize gene expression changes between stem tissues of sweet and grain sorghum have also been conducted (Calviño et al., 2009). The researchers identified several intriguing candidate genes that correlate with high sucrose content in sweet sorghum, but follow up studies to demonstrate causality have not been reported. The identification of genes that positively regulate sucrose content in grass stems is a high priority for biotechnological approaches to improving biomass feedstocks for biofuels production.

### APPROACHES TOWARD INCREASING STEM SINK STRENGTH

Because of the importance of sucrose stored in the stem for both food and biofuel production, increasing its concentration in stem storage parenchyma cells is a major research objective. There are a number of avenues that researchers could target in order to improve stem sink strength. In addition to selecting sweeter varieties (reflecting higher content of sugar in the stem), one approach is to increase stem size, which would mirror an increase in cell number and/or an increase in individual cell expansion. Moreover, there is the potential to increase surface/unloading area for sugars into stem storage parenchyma cells. This is the theory behind breeding efforts that have targeted and successfully led to thicker stems, higher stem juice volume, and increased sucrose concentration in the internodes of both sugarcane and sweet sorghum (Glassop et al., 2007; Slewinski, 2012; Patrick et al., 2013).

Another approach to increasing sucrose content in stems is by reducing the sink strength of other organs (e.g., grains) and thereby reducing competition for photoassimilates. This approach could be significant because competition among sink organs is known to be higher when supply is limited (Marcelis, 1996; Andersen, 2003). Breeding efforts have led to the generation of sweet sorghum varieties with larger stems and reduced panicle size. However, other sorghum lines have been reported to have high stem sugar concentration and still maintain seed yields comparable to those of grain sorghum (Qazi et al., 2012). At the opposite extreme, enhanced sink strength was engineered by removing all but one of the source leaves from sugarcane plants. This manipulation of the sink/source ratio in sugarcane provided evidence for source limitation under high sink demand (McCormick et al., 2006). These data also demonstrated active communication and influence on source activity by sinks tissues, namely, photosynthetic rate increased in the one remaining source leaf to provide sufficient photoassimilates to meet sink demands. Hence, supply and demand equilibrium is important to overall productivity; therefore, targeting one aspect alone might not lead to increased sugar content (McCormick et al., 2009). Thus, there is a great need to study the control of carbon flux in stems, the functions of the genes responsible, and also to determine the limiting factors/enzymes that can be targeted for manipulation in order to achieve the goal of enhancing endogenous sugar content in stem tissues.

Along these lines, caution is warranted when using a transgenic approach to improve stem sugar content by targeting a sucrose metabolizing enzyme, since these efforts have not yet met with envisioned success. Down-regulation of neutral INV in transgenic sugarcane resulted in increased concentrations of stored sucrose and reduced respiration; however, there was a severe negative effect on the overall plant vigor (Rossouw et al., 2010). Further, there was no increase in sucrose levels by constitutively expressing a SPS gene in sugarcane (Vickers et al., 2005). This may suggest that SPP was limiting and that both enzymes need to be coordinately up-regulated to achieve increases in sucrose content. Still, recent breakthroughs have been attained by manipulating sugar levels in sugarcane stems through transgenic approaches. Sugarcane expressing a sucrose isomerase enzyme in stem vacuoles resulted in plants with “near-complete” conversion of sucrose in mature internodes and production of novel metabolites (Wu and Birch, 2007, 2010), although some of the sugar increases attained in greenhouse conditions were not realized in the field (Basnayake et al., 2012; Patrick et al., 2013).

Another possible approach is the manipulation of sucrose transporters, such as SUTs or SWEETs, which could lead to an enhanced sucrose flow from phloem tissues into stem storage cells, particularly in sweet sorghum (**Figure 1C**). However, because of the limited data on the function of the known transporters (Braun and Slewinski, 2009), more work is needed before targeting these membrane proteins for genetic improvement in sugarcane or sweet sorghum. For example, the heterologous overexpression of a spinach (*Spinacia oleracea*) SUT in potato resulted in tubers with increased sugar content and reduced amino acid levels, but yielded tubers with very minimal changes in the starch content and biomass (Leggewie et al., 2003). Further, as SWEET genes have been identified as the targets of microbial pathogens to obtain sugars from plants (Chu et al., 2006; Yang et al., 2006; Chen et al., 2010; Baker et al., 2012; Yuan and Wang, 2013), novel strategies will need to be employed in targeting these genes for genetic engineering to avoid creating highly susceptible crops (Li et al., 2012). As another potential approach for genetic engineering, SUTs have been reported to be regulated by protein phosphorylation (Ransom-Hodgkins et al., 2003; Niittyla et al., 2007). Unfortunately, the responsible protein kinases and protein phosphatases are still unknown. In a different approach, overexpression of TMT1 in *Arabidopsis thaliana *resulted in plants with increased activity in photosynthesis-related genes, decreased sugar use for respiration, and increased capacity for sugar export from source leaves (Wingenter et al., 2010). Hence, TMT1 may represent an attractive molecular target. To our knowledge, the strategy of manipulating the functions of sucrose transporters using transgenic approaches in crops that accumulate sucrose in their stems, such as sugarcane and sweet sorghum, has not been reported.

Because of the economic importance of stored sucrose in the stem, increasing its yield presents a valuable target for plant biotechnologists. However, as indicated above, stem sucrose improvement using transgenic approaches is very challenging. A successful and highly efficient transformation protocol for sweet sorghum is yet to be designed, and in the case of sugarcane, transformation is difficult due to the high rate of gene silencing (Mudge et al., 2009; Merwe et al., 2010). Significant improvement of sugarcane have been reached using conventional breeding, but this is challenging due to its complex polyploid genome (Waclawovsky et al., 2010). Nevertheless, there is potential to increase sugar levels in the stem through metabolic engineering, but we need to expand our understanding of plant sugar metabolism and transport before undertaking such an effort (Patrick et al., 2013). The tantalizing possibility of further improving sweet sorghum and sugarcane stem sucrose content is at hand if we can understand the mechanisms that regulate stem sink strength.

## MOLECULAR CONTRIBUTORS TO STRONG SINK STRENGTH IN GRAINS

**FIGURE 2:**
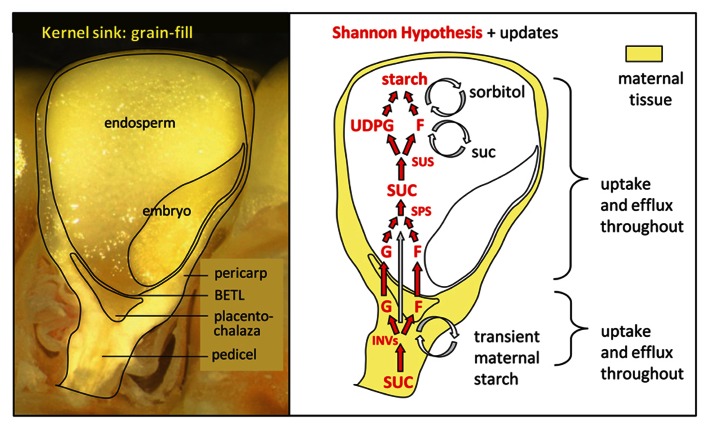
**Maize kernel sink strength during grain fill**. The left panel shows a fresh, longitudinal view of a maize kernel during active starch deposition in the upper endosperm and lower pericarp (12 days after pollination). The path of assimilate entry into endosperm or embryo includes mandatory movement through the cell wall space (apoplast), because these tissues are physically isolated from the maternal structures around them by a lack of plasmodesmatal (symplastic) continuity. The right panel diagrams major contributors to sink strength relative to sites of their spatial distribution in kernels at this stage. The original Shannon hypothesis is shown in red, with updates in black. Maternal tissues are shown in yellow to emphasize the role of apoplastic transfer. Sucrose (SUC) is first cleaved by vacuolar and cell wall invertases (INV) operating in the pedicel, the placenta-chalaza, and especially by cell wall INV in the basal endosperm transfer layer (BETL). Updates to the first portion of this classical path include continual influx and efflux from maternal cells (via transporters and effluxers), and a prominent role for transient starch reserves in maternal tissues (especially the lower pericarp). Assimilates next enter the endosperm across the BETL primarily as hexoses, glucose (G) and fructose (F), but also as SUC. SUC is also resynthesized in basal portions of the endosperm by sucrose phosphate synthase (SPS), and transported to upper regions of the endosperm. Recent evidence shows extremely low oxygen levels in endosperm, which can affect several aspects of kernel sink strength. These include a limitation to metabolism of stored assimilates, advantages of sucrose resynthesis and cleavage by sucrose synthase [SUS (typically cleaving sucrose *in vivo*)], and cycling of sorbitol as a mechanism of redox balance under deep hypoxia. UDPG, uridine-di-phosphoglucose.

The maize kernel during starch-deposition has long been a system of focus for analysis of carbon movement into grains (Sabelli and Larkins, 2009). A classical model for kernel sink strength is outlined by the Shannon hypothesis for cleavage and resynthesis of sucrose entering the maize endosperm (**Figure 2**; Shannon, 1972). In this scenario, sucrose is metabolized by a combination of cell wall and vacuolar INVs in the phloem-unloading zone, and resynthesized in the endosperm prior to starch biosynthesis (Shannon, 1972; Shannon and Dougherty, 1972; Felker and Shannon, 1980). The model is supported by diverse lines of evidence extending from radiotracer studies (Shannon, 1972; Felker and Shannon, 1980), to enzyme activity (Shannon and Dougherty, 1972; Im, 2004; Koch, 2004), mRNA levels (Koch, 1996; Xu et al., 1996), mutant phenotypes (Vilhar et al., 2002; Keeling and Myers, 2010), and environmental perturbations (Andersen et al., 2002; Setter and Parra, 2010). A prominent role for INV in basal regions of the kernel is consistent with proposed advantages to phloem unloading that would enhance overall turgor gradients and pressure-driven movement of sucrose in the phloem (Turgeon, 2010). INV action in this zone has also been proposed to enhance sugar signals important to establishment of young sinks (see below). Although sucrose cleavage is not required for sucrose entry into the developing endosperm (Schmalstig and Hitz, 1987), INV clearly has a major role in the initial metabolism and signaling of sugars entering kernels (Koch, 2004; Ruan et al., 2010; Aoki et al., 2012).

The path of sucrose transfer and metabolism in kernels and other grains has thus emerged as a central feature of their sink strength. Disruptions to any of the steps involved can have major consequences for the capacity to import and use sucrose. **Figure 2** diagrams a current view of maize kernel sink strength. Evidence for each of their roles includes a range of genetic, transgenic, and environmental alterations, beginning with decreases in activities of the sucrose-metabolizing vacuolar INV (Andersen et al., 2002) and cell wall INV (Vilhar et al., 2002) that severely curtail kernel development. Neutral INV involvement has been enigmatic (Huang et al., 1992). Overall assimilate import is also reduced by disruptions to the second path of sucrose cleavage mediated by the reversible SUS reaction (Koch, 2004; Aoki et al., 2012). Sucrose resynthesis in endosperm is well-documented (Shannon, 1972; Hennen-Bierwagen and Myers, 2013), and evidence supports contributions by SPS and SPP to sink strength of grains (Im, 2004; Sharma et al., 2010) and other fruits (Nguyen-Quoc et al., 1999).

Final storage products are the ultimate sinks, and numerous mutations to genes for starch biosynthesis show that this process affects not only the composition of grains and kernels, but also their sink strength (Creech, 1965; Hennen-Bierwagen and Myers, 2013). In rice (*Oryza sativa*), starch biosynthesis is suppressed and amylopectin structure altered if a negative regulator of starch synthesis, *Rice Starch Regulator1* (*RSR1*), is overexpressed. Conversely, when *RSR1* is down-regulated, starch production increases, seed size rises, and overall yield is also greater (Fu and Xue, 2010). Disruption to starch biosynthesis typically elevates sugar levels [as in sweet corn (Creech, 1965; Hennen-Bierwagen and Myers, 2013)], which has far-reaching effects on sugar signals to other metabolic and developmental programs (Koch, 1996, 2004; Ruan et al., 2010; Aoki et al., 2012). Genetic analyses of such mutants in barley (*Hordeum vulgare*), for example, revealed that alteration of ADP-glucose pyrophosphorylase (AGPase) was accompanied by co-regulation of multiple genes for starch biosynthesis, glycolysis, amino acid storage, and sugar/ABA sensing (Faix et al., 2012). In this way, reduced flux into starch biosynthesis led to induction of mechanisms for decreasing sugar accumulation (and accompanying potential for oxidative stress; Faix et al., 2012).

Minimally understood, but potentially influential components of the post-phloem path into kernels are transporters and efflux constituents (Braun, 2012; Patrick et al., 2013). Their involvement is implicated by apoplastic steps of post-phloem transfer in maize kernels, but symplastic roles of these transporters can also include vesicle-based transfer processes (Echeverría, 2000; Lalonde et al., 2004), transient storage by vacuoles (Eom et al., 2011), cellular efflux (Lalonde et al., 2004; Chen et al., 2010, 2012; Baker et al., 2012; Braun, 2012), or action as sensors (Kang and Turano, 2003; Lalonde et al., 2004; Slewinski, 2011; Aoki et al., 2012; Price et al., 2012). In maize kernels, prominent contributions are indicated for both sucrose and hexose transporters (Aoki et al., 2012), and the importance of SWEET-mediated efflux to diverse sinks (Chen et al., 2010, 2012), led to suggested roles in efflux to seeds (Braun, 2012). Maize SWEETs include paralogs expressed most prominently in the basal endosperm transfer layer (BETL; Xiong et al., 2011).

Other potential determinants of sink strength in kernels include not only genes for diverse aspects of both carbon and nitrogen metabolism (Koch, 1997; Coruzzi and Bush, 2001; Uribelarrea et al., 2004; Seebauer et al., 2010) but also those affecting cell wall biosynthesis and expansion (Shevell et al., 2000; Lizana et al., 2010; Manavski et al., 2012; Liu et al., 2013). Although starch is a prominent end-product of assimilates imported into maize kernels, so too is the oil stored in the embryo scutellum (Rolletschek et al., 2005; Shen et al., 2010). Embryo size, its oil content, and carbon partitioning between embryo and endosperm can all be altered at the genetic level in maize (Shen et al., 2010; Yang et al., 2010). Oil biosynthesis and deposition can thus be a significant contributor to strong sink strength in maize kernels. The role of nitrogen in carbon sink strength of kernels may be more complex, since formation of storage proteins can vary in its relation to starch deposition (Singletary et al., 1990; Kang and Turano, 2003; Uribelarrea et al., 2004; Wang et al., 2011). An interface between carbon/nitrogen import is also evident at other levels, including amino acid sensing (Kang and Turano, 2003; Seebauer et al., 2004, 2010), often reciprocal responses to carbon and nitrogen abundance by individual genes (Koch, 1997), limitations to capacity for carbon import in female florets of nitrogen-deficient plants (Peng et al., 2012), and a range of nitrogen effects on kernel sinks (Singletary et al., 1990; Uribelarrea et al., 2004).

A previously unexpected role of low oxygen has emerged for sink strength of maize endosperm, which normally has very low oxygen levels (Rolletschek et al., 2005). Deep hypoxia appears to be a common feature of starch-storing endosperms such as that of barley (Rolletschek et al., 2011), but not of the adjacent embryos (Rolletschek et al., 2005). If this deep hypoxia is indeed characteristic of diverse grain endosperms, then deposition of starch and protein in the world’s major grain crops is likely to occur in a very different endogenous environment than was recognized earlier. Although the outermost endosperm cells are partially oxygenated in smaller grains (Rolletschek et al., 2011), oxygen levels drop to limits of detection immediately inside the pericarp of maize kernels (Rolletschek et al., 2005). Metabolite analyses and modeling studies of kernels concur with predicted, low oxygen alterations to glycolysis, redox status, and other effectors of sink strength (Koch et al., 2000; Méchin et al., 2004; Rolletschek et al., 2005; Grafahrend-Belau et al., 2009; Rolletschek et al., 2011). Implications extend to pyrophosphate (PPi) cycling, and its potential role in balancing starch deposition with glycolytic rate. In a low oxygen endosperm, ATP limitations would favor PPi-driven glycolysis [via PPi-phosphofructokinase (Huber and Akazawa, 1986; Zeng et al., 1998; Koch, 2004)], and this demand for PPi (together with that of UDP-glucose pyrophosphorylase) could be met and balanced by the PPi produced during starch biosynthesis by AGPase. Deep hypoxia in endosperm, rather than being a stress, may thus be advantageous to starch biosynthesis and ultimate sink strength.

**FIGURE 3:**
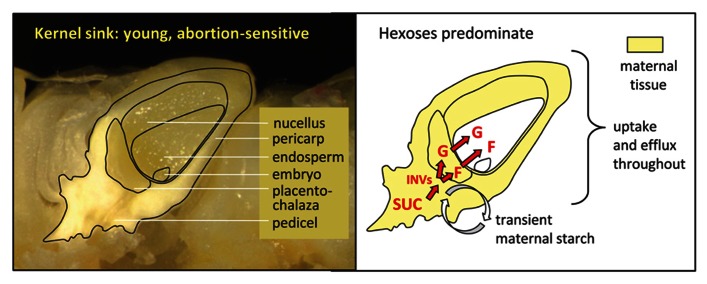
**Maize kernel sink strength in young, abortion-sensitive ovaries**. The left panel shows a fresh, longitudinal view of a maize kernel at eight days after pollination, when maternal tissues predominate in this sink structure. Note the importance of the nucellus and expanding pericarp (fruit wall). The right panel diagrams major contributors to sink strength relative to their spatial distribution. Maternal tissues are shown in yellow to emphasize their dominant role in sink strength, and control over abortion and kernel set at this stage. Like the more mature kernels, sucrose (SUC) in young ovaries is first cleaved by vacuolar and cell wall invertases (INVs) in the pedicel and the placenta-chalaza, and to some extent the newly forming basal endosperm transfer layer. Updates have indicated that collective effects of these INVs are most pronounced in very young kernels, where the hexoses have signaling roles in the cell cycle, cell division, and cell number that markedly enhance ultimate sink strength. Another component of young-kernel sink strength is transient maternal starch, thought to aid maintenance of non-aborting kernels. In addition, continuous efflux and influx of sugars occur throughout maternal tissues, even prior to pollination, highlighting the roles for transporters and effluxers during early development of sink strength in maize kernels. G, glucose; F, fructose.

Sink strength of newly forming kernels is determined differently (**Figure 3**), and can affect which will abort and which will not. The first third of kernel development is vital to establishment of kernel number, since this is the period when maternal “decisions” are made regarding seed-sink load (Setter and Parra, 2010). Until this stage, a young maize grain is largely maternal tissues (nucellus and pericarp) that import sucrose by mechanisms distinct from those of more mature grains (endosperm and embryo; Sabelli and Larkins, 2009; Aoki et al., 2012). These maternal cells of young maize kernels and other grains are symplastically continuous, actively dividing, and rapidly expanding, yet imported assimilates move into both the apoplast and symplast of pre-pollination ovaries (Sabelli and Larkins, 2009; Aoki et al., 2012; Tang and Boyer, 2013). Both vacuolar and cell wall INVs are prominent in provision of hexoses in these organs, not only for downstream carbon metabolism, but also for the hexose-based sugar signals important to up-regulation of sink strength in young organs (Andersen et al., 2002; Koch, 2004; Ruan et al., 2010). Effects include genes for sucrose import, cell cycle regulation, and establishment of new sinks (Koch, 2004; Ruan et al., 2010). Many of the mutations responsible for the most devastating kernel phenotypes are ones that alter sink strength at this early stage of development, through disruption of carbon use in respiration (Manavski et al., 2012; Liu et al., 2013), cell wall biosynthesis (Shevell et al., 2000), or sugar signals to other genes (Koch, 2004; Ruan et al., 2010; Aoki et al., 2012).

Molecular features of strong sinks in small-grain pooid species may well differ, due to the distinctive features of this group compared to the panicoids. The panicoids (e.g., maize, sugarcane, and sorghum) typically use C_4_ photosynthesis and have a maize-type grain structure, whereas the pooids [e.g., rice, wheat (*Triticum aestivum*), and barley] are C_3_ species with a rice-type grain structure. Although not universal, overall source–sink relations in pooids often show a trade-off between seed size and seed number that implies a source-limited system (Acreche and Slafer, 2006). The same appears less evident for C_4_ panicoids such as maize, however, which are typically large, with high capacities for carbon assimilation (Borrás et al., 2004; McCormick et al., 2009). The panicoid and pooid grain structures also differ (Hands et al., 2012), and studies of assimilate entry into them are consistent with different contributions by key determinants of sink strength (Rolletschek et al., 2005, 2011; Aoki et al., 2012).

## MOLECULAR BASIS FOR STRONG SINK STRENGTH IN OTHER FRUITS, SEEDS, AND FLOWERS

The biosynthesis and regulation of cell walls may be central to strong sinks in many organs*. *Young structures in particular, often have high rates of cell division (Boisnard-Lorig et al., 2001) that involve significant allocation of carbon to biosynthesis of new cell walls. Later, during cell expansion, sink strength can also be affected by regulation of cell wall properties, such as the combined relaxation and new synthesis that facilitate turgor-driven expansion (Cosgrove, 2005). This aspect of sink strength can be especially important for accommodating phloem water in minimally transpiring sink organs. Cell wall loosening signals and enzymes can thus be critical to a tissue’s sink strength. Various cell wall enzymes and proteins have been implicated in the loosening that occurs during cell growth, including expansins (Fleming et al., 1997; Link and Cosgrove, 1998) and xyloglucan endotransglucosylase/hydrolases (Fry et al., 1992).

In addition to their structural roles, non-cellulosic cell wall polysaccharides can also be major storage constituents in many fruits and seeds (Buckeridge, 2010). In this respect their contribution to sink strength may be analogous to that of starch. Examples include the xyloglucans in endosperms of legumes such as nasturtium (*Tropaeolum majus*; Edwards et al., 1985), the galactomannans in seeds such as fenugreek (*Trigonella foenum-graecum*; Reid and Meier, 1972; Reid et al., 1977), and the β-glucans and arabinoxylans that appear early in developing endosperms of wheat and barley (Meier and Reid, 1982; Philippe et al., 2006). In the starchy endosperm of barley, β-glucans represent 70% of wall material at maturation (Fincher, 1975; Wilson et al., 2006), and together with arabinoxylans [which comprise 85% of the aleurone wall (McNeil et al., 1975)], provide 18.5% of the carbon used by germinating embryos (Morrall and Briggs, 1978). The sink strength of developing seeds, as well as their subsequent germination, may also be aided by the water-holding features of arabinoxylans [100 g H_2_0 g^-1^ polymer (Izydorczyk and Biliaderis, 1995)]. This 100-to-1 ratio of water-to-polysaccharide (g/g) indicates a capacity for arabinoxylans to aid handling of the excess water entering sinks that have reached their full size and are no longer transpiring (this can otherwise affect assimilate import; Koch and Avigne, 1990; Huang et al., 1992; Zhang et al., 2007; Turgeon, 2010).

Diverse molecular contributors to sink strength emerge in analyses of different plant systems. Overall import of photosynthates can be increased in a number of structures and species, for example, by up-regulating genes for starch biosynthesis (Wang et al., 2007; Baroja-Fernández et al., 2009; Li et al., 2011, 2013). Also, cell cycle regulation can make important differences to sink strength, as evidenced by overexpression of a D-type cyclin gene in developing seeds of Arabidopsis. Resulting seeds were larger, cell number and size were greater, and growth was increased for both embryo and endosperm (Collins et al., 2012).

Strong floral sinks are vital for establishment of the fruits they will become. Rapid cell division and expansion are critically important to virtually all floral parts, and especially the structures involved in pollination (Xu et al., 1996; Cosgrove, 2005; Aoki et al., 2012; Reeves et al., 2012). Where showy petals are produced, INVs often determine blossom size and floral sink strength (Gübitz et al., 2009). Nectary structures also vary markedly, but again, involve genes for INV, vacuolar compartmentalization, and secretory functions (Ge et al., 2000; Gübitz et al., 2009; Chen et al., 2010). Pollination success in maize requires an expansin- and INV-mediated elongation of the floral silk (a stigmatic style), which is one of the most rapidly elongating structures in the plant kingdom (Xu et al., 1996; Cosgrove, 2005). Similar INV requirements are evident for anthers of rice (Ruan et al., 2010). Expansion and sink strength of pre-pollination ovaries also depend on INVs that are sugar- and stress-sensitive determinants of later sink strength (Xu et al., 1996; Andersen et al., 2002; Koch, 2004; Lizana et al., 2010). Transgenic approaches to increase maize yields are also targeting the enhancement of INV activity and/or starch biosynthesis in florets (Tomes et al., 2011). These centrally important aspects of expansion are also mediated by auxin, which is critical to the earliest stages of sink strength development in flowers (Barazesh et al., 2009; Phillips et al., 2011; Reeves et al., 2012).

Expansion is also a prominent contributor to sink strength of fleshy fruit, regardless of whether a given fruit stores starch or soluble sugars. The strongest sinks among young fruit typically have the highest INV activity, greatest respiration rate, and most rapid cell division (Huang et al., 1992; Koch, 2004; Ruan et al., 2012b). Central contributions have also been attributed to sugar transporters (Zhang et al., 2007; Slewinski, 2011; Aoki et al., 2012), and are suggested for SWEET-mediated efflux in fruit (Baker et al., 2012; Braun, 2012). Roles of auxin and gibberellins parallel those noted above for floral sinks (Aoki et al., 2012). Water handling becomes a key feature for those organs importing sugars without transpiring or expanding [e.g., citrus fruit during most of their development (Koch and Avigne, 1990; Huang et al., 1992)], since excess phloem water can become a problem for such sinks (Huang et al., 1992; Zhang et al., 2007; Turgeon, 2010).

## FEAST-OR-FAMINE SIGNALS AND FEED-FORWARD ENHANCEMENT OF SINK STRENGTH

Sugar-responsiveness is a prominent feature of genes contributing to sink strength of developing organs, and provides an important mechanism for sink adjustment to source supplies (Koch, 1996; Koch et al., 2000; Ramon et al., 2008; Ruan et al., 2010; Tiessen and Padilla-Chacon, 2013; Xiong et al., 2013). Sink genes up-regulated by sugars span the full spectrum of those involved in sucrose import and use. They range from sucrose cleavage to deposition of storage products (Li et al., 2002; Smidansky et al., 2002; Ramon et al., 2008; Kang et al., 2010). Similar regulation occurs at multiple levels from transcription (Koch, 1996, 2004; Li et al., 2002; Ramon et al., 2008) to translation (Zeng et al., 1998), and from mRNA longevity (Zeng et al., 1998; Koch, 2004; Huang et al., 2007) to protein turnover (Koch, 1996, 2004; Huang et al., 2007). Multiple paths of sugar signaling are also involved, such that sucrose can be sensed differently from hexoses, and endogenous metabolic flux can be sensed differently than exogenous sugars (Koch, 1996; Ruan et al., 2010; Tiessen and Padilla-Chacon, 2013). The end result is a means by which strongest sinks can be maximally up-regulated through input from greatest photosynthate supplies [The reverse would also allow a balanced down-regulation (Kang et al., 2010)].

Still further enhancement of sink strength can occur through an INV-mediated, feed-forward process that increases hexose production, and thus those sugar signals that arise from hexose-based sensing (Koch, 1996, 2004; Huang et al., 2007; Ruan et al., 2010; Tiessen and Padilla-Chacon, 2013). Multiple hexose-based avenues of sugar signaling are known effectors of genes for the cell cycle, hormone interaction, and others important to development of new sinks (Koch, 1996, 2004; Rolland et al., 2006; Ramon et al., 2008; Kang et al., 2010; Ruan et al., 2010; Moghaddam and Van den Ende, 2013). The same process may also be involved in initiation of new sinks through demonstrated interactions with phytochrome sensing (Kang et al., 2010; Moghaddam and Van den Ende, 2013), low oxygen signals (Koch et al., 2000; Koch, 2004), and hormonal control of meristem fate (Francis and Halford, 2006). In developing seeds and fruits, a predominance of hexoses is often associated with cell division and expansion, whereas elevated sucrose levels coincide with differentiation and maturation (Koch and Avigne, 1990; Weber et al., 1997; Sabelli and Larkins, 2009; Ruan et al., 2012b).

These changes are compatible with a “feast-or-famine” model for adjustment of source–sink relations in plants (Koch, 1996; Coruzzi and Bush, 2001; Mandadi et al., 2009), a framework initially proposed for understanding sugar responses by multicellular organisms (Koch, 1996). A highly conserved, ancient system is involved, and in microorganisms it constitutes an essential means of sensing and acclimating to the nutrient environment (e.g., the *lac* operon and others; Yokoyama et al., 2006). Maximal sink strength of plant organs will thus include “feast” genes that respond to carbohydrate abundance and enhance its use. Similarly, source capacity will increase when enhanced sink demands for photosynthate reduce sugar levels in leaves and up-regulate expression of “famine” genes for acquisition of the limited carbon resources (e.g., de-repression of photosynthetic genes). Indirect effects of enhanced sink strength therefore include higher rates of photosynthesis in diverse systems due to release of feed-back inhibition at a metabolic level and gene repression at a transcriptional level (Koch, 1996, 2004; Ramon et al., 2008; Kang et al., 2010). Greater overall productivity and yield is thus a reasonable prediction for instances where sink strength can be increased.

## CONCLUDING REMARKS

Increased demand for food and energy security compels researchers to investigate different avenues to improve plants to meet our needs. Carbohydrates stored in non-photosynthetic sink organs are vital to humans as food, feed, fuel, and for other industrial uses; therefore, understanding the functions of the molecular drivers of sink strength is essential to enhance the capability of sink organs to import photoassimilates. Some of the sucrose transport pathways and important players for carbohydrate storage and utilization have been identified. However, future research should focus on examining and understanding the exact roles of different sugar transporters, metabolic control of carbohydrate storage in sinks, sugar signaling regulating sink strength, and long-distance communication coordinating carbohydrate partitioning between sink and source tissues. For example, it is not clear how sucrose transporters, such as SWEETs, contribute to sink strength. Furthermore, it remains to be shown whether their genetic manipulation will enhance the delivery of photoassimilates to grains, seeds, and fruits for food, or, in the case of sweet sorghum and sugarcane, to stems for biofuel production. Detailed genetic, biochemical, and physiological analyses, in addition to molecular and genomic investigations, will facilitate the identification and characterization of novel players involved in defining sink strength, and will provide a deeper knowledge of how plants regulate whole-plant carbohydrate partitioning.

## Conflict of Interest Statement

The authors declare that the research was conducted in the absence of any commercial or financial relationships that could be construed as a potential conflict of interest.
